# Association of asthma comorbidity with poor prognosis of coronavirus disease 2019

**DOI:** 10.1016/j.waojou.2021.100576

**Published:** 2021-08-16

**Authors:** Sae-Hoon Kim, Eunjeong Ji, Seung-Hyun Won, Jungwon Cho, Yong-Hyun Kim, Soyeon Ahn, Yoon-Seok Chang

**Affiliations:** a Department of Internal Medicine, Seoul National University Bundang Hospital, Seongnam, South Korea; b Institute of Allergy and Clinical Immunology, Seoul National University Medical Research Center, Seoul National University College of Medicine, Seoul, South Korea; c Department of Internal Medicine, Seoul National University College of Medicine, Seoul, South Korea; d Division of Statistics, Medical Research Collaborating Center, Seoul National University Bundang Hospital, Seongnam, South Korea; e Department of Pharmacy, Seoul National University Bundang Hospital, Seongnam, South Korea

**Keywords:** Asthma, COVID-19, Exacerbation

## Abstract

**Background:**

While global health agencies have listed asthma as a vulnerability for severe cases of coronavirus disease 2019 (COVID-19), the evidence supporting this is scarce.

**Methods:**

A nationwide cohort study was conducted using the validated Korean national health insurance claim data of patients diagnosed with COVID-19 between January 1 and April 8, 2020. Asthma comorbidity was determined using a diagnosis code assigned by the physician and the prescription of asthma-related medications. The clinical course of COVID-19 was classified into 3 severity grades according to the requirements for oxygen supply and mechanical ventilation. We also evaluated the association of asthma with overall and in-hospital mortality of COVID-19.

**Results:**

Asthma morbidity was a significant risk factor for severe COVID-19 (grade 2 requiring oxygen supply) (adjusted odds ratio [aOR] = 1.341, 95% confidence interval [CI], 1.051−1.711, P = 0.018) and grade 3 requiring mechanical ventilation or leading to death (aOR = 1.723, 95% CI: 1.230−2.412, P = 0.002) multinomial logistic regression adjusting co-risk factors. Asthma was also significantly associated with mortality of COVID-19 (aOR = 1.453, 95% CI: 1.015−2.080, P = 0.041) and was revealed to have a shorter time to in-hospital mortality of COVID-19 (P < 0.001). Patients with recent asthma exacerbation showed more severe COVID-19 of grade 3 (OR = 7.371, 95% CI: 2.018−26.924, P = 0.003) and higher mortality (OR = 9.208, 95% CI: 2.597−32.646, P < 0.001) in univariable analysis, but the statistical significance was not found in multivariable analysis.

**Conclusion:**

Asthma morbidity was associated with severity and mortality of COVID-19. Patients with asthma should pay more attention to avoid worsening of COVID-19.

## Introduction

The novel coronavirus disease 2019 (COVID-19) pandemic caused by the severe acute respiratory syndrome coronavirus 2 (SARS-CoV-2) is a major challenge for global healthcare systems worldwide. The World Health Organization (WHO) has listed airway disease as a vulnerable condition for COVID-19 along with diabetes and heart disease, and the U.S. Centers for Disease Control and Prevention (CDC) announced that moderate to severe asthma may lead to higher risk of serious cases of COVID-19.^1^^,^^2^ COVID-19 may cause pneumonia and acute respiratory diseases and lead to asthma attacks because respiratory viral infections are the most common triggers of asthma exacerbation. Patients with asthma also showed impaired production of antiviral interferons (IFNs).^3^ However, early reports of COVID-19 did not indicate asthma as a risk factor of infection or severe illness with the virus.^4^^,^^5^ Although the evidence for asthma as a risk for COVID-19 is scarce, a contradictory hypothesis that asthma might be protective against COVID-19 has also been suggested.^6^ SARS-CoV-2 uses angiotensin-converting enzyme 2 (ACE2) for cell entry in the airway epithelial cells.^7^ In recent reports, airway epithelial cells from patients with allergy or asthma showed lower expression of ACE2, and ACE2 expression was inversely correlated with type 2 (T2) cytokine levels and T2 signature molecular expression.^8^^,^^9^ Moreover, patients with COVID-19 having poor outcomes showed early robust type I interferon expression together with a high level of IFN-related chemokine and IFN-γ.^10^ Based on these reports, an intriguing clinical question has been raised about whether patients with asthma who have lower production of IFNs and higher T2 cytokines might have better outcomes than healthy individuals.^6^

This study aimed to investigate whether the comorbidity of asthma affects the clinical outcomes of COVID-19 in a retrospective cohort study using nationwide population-based claim data from the Korean National Health Insurance Service (NHIS).

## Methods

### Study population and data sources

A retrospective cohort study was conducted using the Korean NHIS claim data of patients diagnosed with COVID-19 infection in South Korea between January 1 and April 8, 2020. The claims data were provided by the Korean NHIS with anonymized personal identifiable information and contained basic demographic information and information on outpatient clinic visits, hospitalizations, procedures, and prescriptions matched with the list of patients confirmed with COVID-19. All patients were confirmed by a positive PCR test for COVID-19, and the list of these patients was provided by the Korean Centers for Disease Control and Prevention. The data were also linked with mortality data from the Korean Statistical Information Service. The use of the claims data was approved by the NHIS, and the study was approved by the Institutional Review Board. The requirement for informed consent was waived under the approval of the IRB.

### Working definition of asthma and comorbidities

In the confirmed COVID-19 cohort, patients with asthma were selected when the following criteria were fulfilled: diagnosis of asthma by a physician with ICD-10 code J45.x or J46.x and simultaneous prescription of any asthma-related medication at least twice a year during the last 3 years (2017–2019). Asthma-related medications included inhaled corticosteroids (ICS), long-acting β2 agonists (LABA), a combination of ICS and LABA in a single inhaler (ICS/LABA), oral leukotriene antagonists, short-acting β2 agonists, xanthine derivatives, and biologics for asthma such as omalizumab, mepolizumab, and reslizumab. As the claim data did not include detailed clinical information about subjects’ lung function and asthma control status, we evaluated asthma control status indirectly using a history of asthma exacerbations requiring emergency department visits in the last year. Cases of asthma exacerbation were detected from the claims data for the prescription of salbutamol sulfate medication among those claimed by the code of emergency medical care. The comorbidity index was evaluated with comorbid diseases used in the Charlson Comorbidity Index (CCI) criteria, except asthma, and categorized into none and one or more (≥1). Comorbidity data were assessed using the ICD-10 codes recorded during the past year for each patient.

### Assessment of COVID-19 outcomes

The severity of the SARS-CoV-2 infection was classified into 3 severity grades according to the requirements for oxygen supply and mechanical ventilation. Grade 1 was defined as no requirement for oxygen supply. Grade 2 was the requirement of oxygen supply with a nasal prong (M0040), high flow (M0046), or continuous positive airway pressure (MM360, MM400). Grade 3 was defined as the requirement for mechanical ventilation (M0850, M0857, M0858, M0860) with or without extracorporeal membrane oxygenation (ECMO) (O1903, O1904) or death. The outcome of COVID-19 was also evaluated in terms of mortality and time to death in cases of hospital death due to COVID-19.

### Statistical analysis

A *t*-test for continuous variables and chi-squared or Fisher's exact test for categorical variables were used to compare the demographic and clinical characteristics between asthma and non-asthma groups. Univariable analysis was performed using a chi-squared test to compare the severity and mortality of COVID-19 according to the presence or severity of asthma. Multinomial logistic regression analysis was performed to analyze the impact of asthma on the severity and mortality of COVID-19. Potential risk factors included age, sex, residential area, and comorbidities. Analysis of the time to COVID-19 mortality was performed using a log-rank test. Statistical analyses were performed using SAS Enterprise Guide 7.1. (SAS Institute, NC, USA), and R version 3.5.2. A two-sided P-value <0.05 was deemed to indicate statistical significance.

## Results

### Demographic and clinical characteristics of study subjects

**Supplementary Fig. 1:**
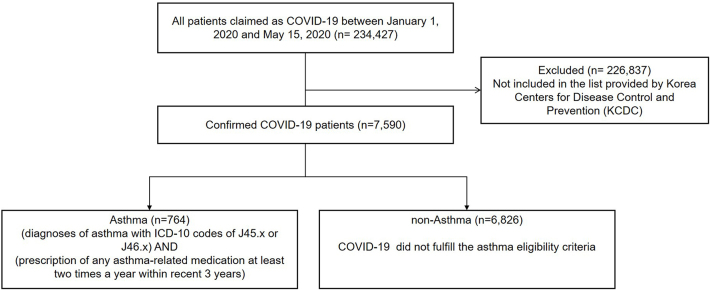
Summary of the study population

A total of 7590 patients diagnosed with COVID-19 between January 1 and April 8, 2020, were enrolled in the study. The mean age was 45.9, and 3095 patients (40.8%) were male. The twenties were the largest age group (24.4%), and elderly over the age of 65 accounted for 18.0% of the study population. The most common area of residence was Daegu/Kyungbuk (54.2%), which was the first endemic area of COVID-19 in South Korea between February and March 2020. The mortality rate of this population was 3.0% in the study period; 2.7% died in hospitals. Patients with any type of comorbidity indicated in the CCI other than asthma, were 49.5%. According to the working definition of asthma, 764 patients (10.1%) were classified into the asthma group, and the others were classified into the control group (Supplementary Fig. 1). The mean age and proportion of female patients were significantly higher in the asthma group than in the control group. The asthma group showed higher rates of residence in Daegu/Kyungbuk and diagnosis in February 2020, the first endemic area and endemic period of COVID-19 in South Korea, respectively. Comorbidities including myocardial infarction, heart failure, cerebrovascular diseases, chronic respiratory diseases other than asthma, diabetes, liver disease, and renal diseases were more common in the asthma group than in the control group (Table 1).

**Table 1:** Demographic characteristics of the study patients.

	Total (n = 7590)	Asthma Cases (n = 764)	Non-asthmatic controls (n = 6826)	P-value
Age	45.87 ± 19.77	50.34 ± 22.32	45.37 ± 19.41	<0.001
Sex (male)	3095 (40.8%)	283 (37.0%)	2812 (41.2%)	0.027
Age group (years)				
<9	82 (1.1%)	40 (5.2%)	42 (0.6%)	<0.001
10−19	349 (4.6%)	27 (3.5%)	322 (4.7%)	
20−29	1852 (24.4%)	105 (13.7%)	1747 (25.6%)	
30−39	777 (10.2%)	72 (9.4%)	705 (10.3%)	
40−49	1008 (13.3%)	93 (12.2%)	915 (13.4%)	
50−59	1502 (19.8%)	131 (17.2%)	1371 (20.1%)	
60−69	1056 (13.9%)	130 (17.0%)	926 (13.6%)	
70−79	591 (7.8%)	100 (13.1%)	491 (7.2%)	
>80	373 (4.9%)	66 (8.6%)	307 (4.5%)	
Elderly (>65 years)	1365 (18.0%)	217 (28.4%)	1148 (16.8%)	<0.001
Region of residence				
Capital area	1861 (24.5%)	148 (19.4%)	1713 (25.1%)	0.001
Daegu, Kyungbuk	4117 (54.2%)	454 (59.4%)	3663 (53.7%)	
Others	1612 (21.2%)	162 (21.2%)	1450 (21.2%)	
Timing of COVID-19 diagnosis				
January 2020	17 (0.2%)	1 (0.1%)	16 (0.2%)	0.017^a^
February 2020	2156 (28.4%)	246 (32.2%)	1910 (28.0%)	
March 2020	5025 (66.2%)	492 (64.4%)	4533 (66.4%)	
April–May 2020	391 (5.2%)	25 (3.3%)	367 (5.4%)	
Major comorbidities				
Myocardial infarction	64 (0.8%)	12 (1.6%)	52 (0.8%)	0.020
Heart failure	269 (3.5%)	54 (7.1%)	215 (3%)	<0.001
Peripheral vascular disease	536 (7.1%)	102 (13.4%)	434 (6.4%)	<0.001
Cerebrovascular disease	488 (6.4%)	71 (9.3%)	417 (6.1%)	<0.001
Chronic pulmonary disease other than asthma	1477 (19.5%)	292 (38.2%)	1185 (17.4%)	<0.001
Diabetes	1052 (13.9%)	148 (19.4%)	904 (13.2%)	<0.001
Liver disease	1365 (18.0%)	191 (25.0%)	1174 (17.2%)	<0.001
Renal disease	98 (1.3%)	16 (2.1%)	82 (1.2%)	0.038
Malignancy	323 (4.3%)	42 (5.5%)	282 (4.1%)	0.073
Comorbidity index				
None (0)	3834 (50.5%)	259 (33.9%)	3575 (52.4%)	<0.0001
One or more (≥1)	3756 (49.5%)	505 (66.1%)	3251 (47.6%)	

^a^ P-value for Fisher's exact test, other P-values were derived from the chi-squared tests

### Association of asthma morbidity with severity and mortality of COVID-19

**Fig. 1:**
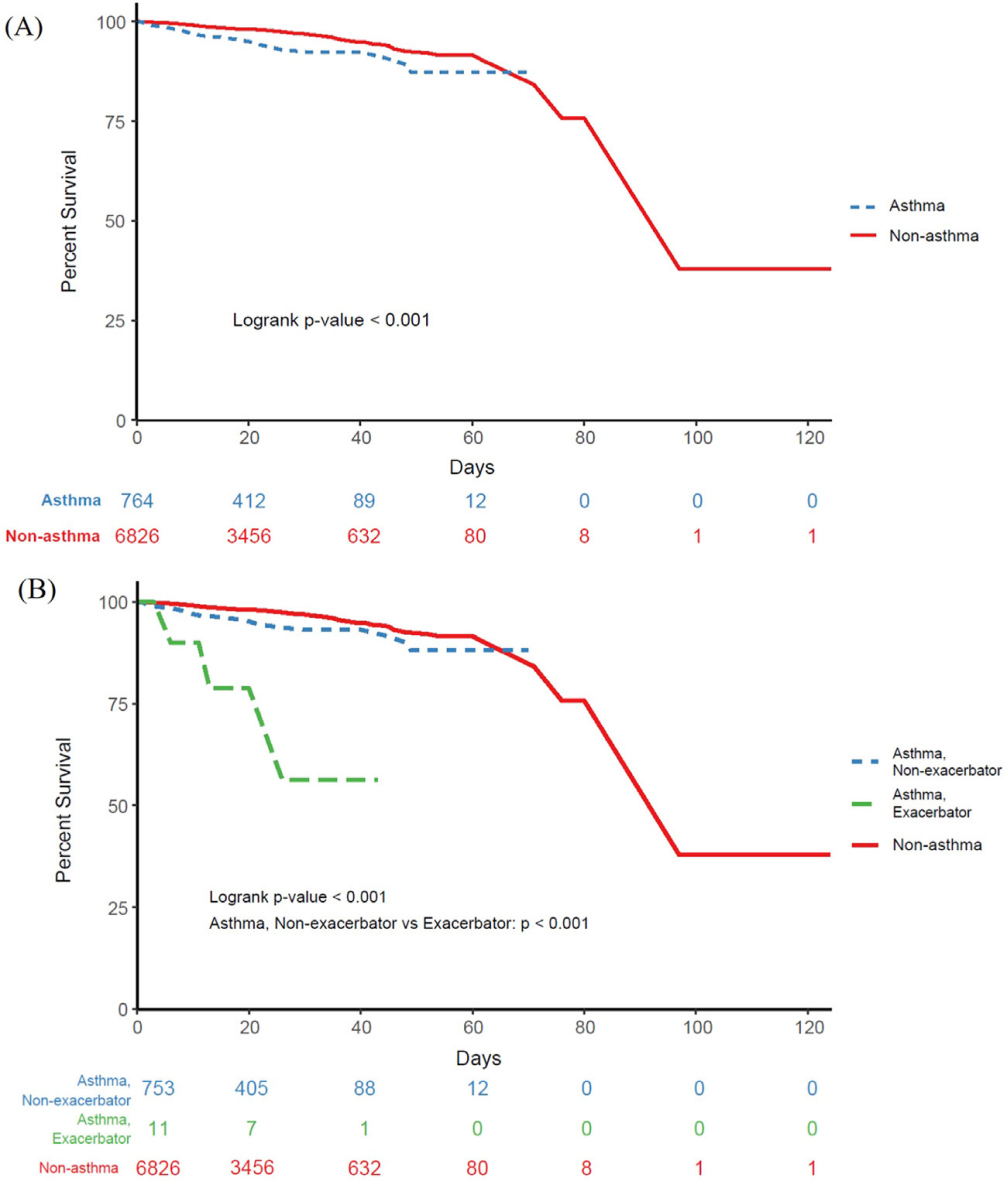
The survival curve for hospital death due to the coronavirus disease 2019 (COVID-19) according to asthma morbidity and recent exacerbation of asthma. (A) Comparison between patients with asthma and the controls, (B) Comparison between patients with asthma with recent exacerbations, those without recent exacerbation, and controls

Patients with asthma showed a more severe form of COVID-19 infection. Grade 2 severity of COVID-19 requiring oxygen therapy and grade 3 severity of COVID-19 requiring mechanical ventilation and leading to death were significantly more prevalent in the asthma group than in the control group. The crude mortality rate was also significantly higher in patients with COVID-19 and asthma than in the controls (6.3% vs. 2.6%, OR = 2.489, P < 0.001) (Table 2). In the multinomial logistic regression analysis to adjust confounding risk factors for the severity of COVID-19 including age, sex, area of residence, comorbidities, and morbidity of asthma was determined as a significant risk factor for severe disease COVID-19 for both grade 2 (adjusted OR [aOR] = 1.341, 95% confidence interval [CI] 1.051−1.711, P = 0.018) and grade 3 (aOR = 1.723, 95% CI: 1.230−2.412, P = 0.002). Asthma morbidity was also significantly associated with COVID-19 mortality, even after adjusting for other risk factors (aOR = 1.453, 95% CI: 1.015−2.080, P = 0.041) (Table 3). In the survival analysis, the presence of asthma was revealed to have a significantly shorter time to in-hospital COVID-19 mortality (P < 0.001) (Fig. 1A).

**Table 2:** Outcomes of COVID-19 according to asthma morbidity

Outcomes	Total (n = 7590)	Asthma Cases (n = 764)	Non-asthmatic Controls (n = 6826)	P-value	Adjusted OR (95% CI)
Severity of COVID-19					
Grade 1	6637 (87.4%)	603 (78.9%)	6034 (88.4%)		
Grade 2	680 (9.0%)	103 (13.5%)	577 (8.5%)	<0.001	1.786 (1.425−2.239)
Grade 3	273 (3.6%)	58 (7.6%)	215 (3.1%)	<0.001	2.702 (1.998−3.653)
Mortality	227 (3.0%)	48 (6.3%)	179 (2.6%)	<0.001	2.489 (1.794−3.455)

**Table 3:** Multivariate analysis for severity and mortality for COVID-19 according to asthma morbidity adjusting risk factors

	Grade 2 of COVID-19		Grade 3 of COVID-19		Mortality	
	aOR (95% CI)	P-value	aOR (95% CI)	P-value	aOR (95% CI)	P-value
Presence of asthma	1.341 (1.051−1.711)	0.018	1.723 (1.230−2.412)	0.002	1.453 (1.015−2.080)	0.041
Old age (≥65 years)	5.153 (4.302−6.172)	<0.001	16.423 (11.928−22.612)	<0.001	18.261 (12.249−27.222)	<0.001
Male sex	1.439 (1.216−1.704)	<0.001	2.091 (1.606−2.721)	<0.001	1.877 (1.413−2.493)	<0.001
Residence in capital area	1.192 (0.884−1.608)	0.249	1.566 (0.854−2.873)	0.147	1.483 (0.698−3.151)	0.305
Residence in Daegu/Kyungbuk area	1.746 (1.379−2.209)	<0.001	3.073 (1.958−4.823)	<0.001	3.488 (2.027−6.003)	<0.001
Presence of comorbid disease (comorbidity index ≥1)	2.274 (1.864−2.775)	<0.001	3.432 (2.278−5.172)	<0.001	3.379 (2.045−5.584)	<0.001

### Association of recent asthma exacerbation with the severity and mortality of COVID-19

Among the 764 patients with asthma, 11 were determined to have recent asthma exacerbation requiring emergency department visits according to our working criteria. Patients with recent asthma exacerbation showed a more severe case of COVID-19, grade 3 requiring mechanical ventilation, (OR = 7.371, P = 0.003) and higher mortality (OR = 9.208, P < 0.001) in the univariable analysis. However, statistical significance was not found in the multivariable analysis adjusting for other risk factors of age, sex, residential area, and comorbidities. In the multivariable analysis, old age was the only independent risk factor for the severity and mortality of COVID-19 infection (Table 4). Patients who had recent asthma exacerbation showed a significantly shorter time to in-hospital death of COVID-19 in the Kaplan-Meier curve of survival analysis (P < 0.001) (Fig. 1B).

**Table 4:** Association of asthma control with severity and mortality of COVID-19.

	Grade 2 of COVID-19		Grade 3 of COVID-19		Mortality	
	OR (95% CI)	P-value	OR (95% CI)	P-value	OR (95% CI)	*p*-value
Crude						
Uncontrolled asthma^a^	0.976 (0.116−8.189)	0.982	7.371 (2.018−26.924)	0.003	9.208 (2.597−32.646)	<0.001
Adjusted						
Uncontrolled asthma^a^	0.347 (0.039−3.102)	0.344	2.390 (0.564−10.124)	0.237	3.617 (0.892−14.678)	0.072
Old age (≥65 years)	5.155 (3.151−8.435)	<0.001	10.105 (4.941−20.699)	<0.001	15.631 (5.767−42.365)	<0.001
Male sex	1.774 (1.123−2.804)	0.014	1.541 (0.845−2.811)	0.158	1.431 (0.753−2.716)	0.274
Residence in capital area	1.784 (0.725−4.393)	0.208	0.626 (0.116−3.398)	0.588	0.993 (0.160−6.161)	0.994
Residence in Daegu/Kyungbuk area	2.607 (1.306−5.203)	0.007	2.595 (1.040−6.476)	0.041	2.345 (0.790−6.956)	0.125
Presence of comorbid disease (comorbidity index ≥1)	2.191 (1.154−4.158)	0.016	2.754 (0.910−8.338)	0.073	2.873 (0.629−13.1214)	0.173

^a^ Comparison of patients with asthma exacerbation requiring ER visits to those without asthma exacerbation in the last year

### Subgroup analysis in the elderly

Since old age was the dominant risk factor for the severity of COVID-19, we additionally performed a subgroup analysis in elderly patients over the age of 65. Here, asthma morbidity was significantly associated with grade 2 (OR = 1.454, P = 0.031) and grade 3 (OR = 1.749, P = 0.004) of severe COVID-19 and mortality of COVID-19 (OR = 1.619, P = 0.012). In the multivariable analysis, asthma morbidity was a significant risk factor for grade 3 disease along with other risk factors including age, sex, residence in Daegu/Kyungbuk, and comorbidity (adjusted OR = 1.563, P = 0.032). However, statistical significance was not found in the multivariable analysis for grade 2 severity and mortality in the subgroup analysis of the elderly (Table 5).

**Table 5:** Subgroup analysis in the elderly (≥65 years): association of asthma morbidity with severity and mortality of COVID-19

	Grade 2 of COVID-19		Grade 3 of COVID-19		Mortality	
	OR (95% CI)	P-value	OR (95% CI)	P-value	OR (95% CI)	P-value
Crude						
Presence of asthma	1.454 (1.035−2.044)	0.031	1.749 (1.192−2.567)	0.004	1.619 (1.113−2.356)	0.012
Adjusted						
Presence of asthma	1.377 (0.974−1.946)	0.071	1.563 (1.039−2.351)	0.032	1.438 (0.963−2.149)	0.076
Old age (≥65 years)	1.034 (1.016−1.053)	<0.001	1.112(1.089−1.137)	<0.001	1.112 (1.089−1.137)	<0.001
Male sex	1.263 (0.971−1.644)	0.082	2.158 (1.555−2.995)	<0.001	2.077 (1.490−2.895)	<0.001
Residence in capital area	1.363 (0.790−2.352)	0.266	1.438 (0.684−3.024)	0.338	1.135 (0.489−2.636)	0.768
Residence in Daegu/Kyungbuk area	1.662 (1.152−2.399)	0.007	1.997 (1.196−3.335)	0.008	2.131 (1.208−3.760)	0.009
Presence of comorbid disease (comorbidity index ≥1)	1.184 (0.824−1.703)	0.361	1.942 (1.105−3.414)	0.021	1.944 (1.058−3.571)	0.032

## Discussion

COVID-19 is a major threat to global health, and asthma is considered a risk factor for severe cases of COVID-19. However, the evidence for the effect of asthma on COVID-19 has not been well demonstrated. Our study revealed that asthma morbidity is associated with more severe disease and mortality of COVID-19. These findings were demonstrated even in the multivariable analysis adjusting for other confounding risk factors such as age, residence in endemic areas, comorbidities, and subgroup analysis confined to the elderly. Moreover, patients with uncontrolled asthma who had experienced asthma exacerbations requiring emergency department visits in the past year showed a greater risk of severe COVID-19, higher mortality rate, and shorter time to in-hospital mortality of COVID-19, although the uncontrolled asthma was not independent risk factor in the multivariate analysis. Our results indicate special caution for severe cases of COVID-19 in patients with asthma, especially in those with a history of recent asthma exacerbation.

The COVID-19 pandemic is an ongoing global health issue. Until December 2020, more than 79 million COVID-19 cases have been reported with over 1.7 million deaths worldwide.^11^ SARS-CoV-2 is a respiratory pathogenic virus, and whether the morbidity of asthma, a common respiratory disease, affects COVID-19 in terms of disease incidence and severity is under debate. Early studies have not indicated a high prevalence of asthma in patients with COVID-19. According to China, no patient with COVID-19 was reported to have asthma as a comorbidity.^4^^,^^5^ A study from Italy also reported a low prevalence of asthma (<3%).^12^ However, a recent report from the CDC showed a high prevalence of asthma in patients hospitalized with COVID-19 in the United States. Asthma was present in about 17% of all admitted patients and its prevalence was as high as 27% in the 20−49 years age group.^13^ In our study population, the overall prevalence of asthma (according to our working definition) was 10.1%, which is higher than the prevalence reported in the Korean general population. This seems to be related to our definition of asthma, which includes patients with a history of asthma treatment within the last 3 years. The prevalence of current asthma in patients who had an asthma diagnosis with simultaneous asthma medication history in the past year was 5.3% (data not shown); this was similar to previous reports of asthma prevalence in the general population.^14^ It is unclear whether a patient with asthma is more susceptible to acquire COVID-19. Large-scale studies of the prevalence of asthma in patients with COVID-19 or the incidence of COVID-19 in patients with asthma compared with the control group are needed in the future.

Our study indicates a poor prognosis and higher mortality of COVID-19 in patients with asthma. One study reported that asthma was significantly associated with longer intubation time and a trend of longer hospitalization period.^15^ However, data on the outcomes of COVID-19 related to asthma morbidity have been scarce despite theoretical suggestions of the association. How asthma affects the prognosis of COVID-19 is an intriguing clinical topic, and several mechanisms have been suggested for the explanation of the interaction between the 2 diseases. ACE2 acts as a cellular receptor for SARS-CoV-2, enabling acute viral replication.^7^ Early studies showed lower expression of ACE2 in airway epithelial cells of patients with allergies or asthma under T2 inflammation, suggesting a lower incidence and better outcomes of COVID-19 in patients with asthma.^8^^,^^9^ Patients with chronic obstructive pulmonary disease had increased ACE2 protein expression, whereas those with asthma had decreased expression of ACE2 protein in lower airway tissues. T2 cytokines such as IL-4 and IL-13 were downregulated, but TNF-a, IL-12, and IL-17A upregulated ACE mRNA expression in BEAS-2B cells.^16^ However, the gene expressions of ACE2 and transmembrane serine protease 2 (TMPRSS2) were similar in asthma and healthy controls in recent studies using samples from a severe asthma research program-3 (SARP-3) population.^17^ A recent study showed higher expression of ACE2-related genes in bronchial biopsy, bronchoalveolar lavage, or blood. In addition, higher expression of ACE2-related genes in the lesioned skin of patients with atopic dermatitis was noted.^18^ ACE2 expression was linked with viral response genes related to severe cases of COVID-19 in patients with T2 low asthma.^19^ ACE2 expression in patients with asthma remains controversial. Prior reports have shown that among different endotypes of asthma, it is the non-allergic asthma which is linked to prolonged need for intubation.^20^^,^^21^ Non-allergic asthma was associated with higher risk of severe COVID-19, while atopy is a potential protective and positive prognostic factor for decreased severity of COVID-19.^20^^,^^22^ The phenotype of asthma, for example, allergic or nonallergic, childhood-onset, or adult-onset, might affect the acquisition and outcome of COVID-19 in connection with ACE2 expression.

Regarding another mechanism of interaction between asthma and COVID-19, patients with asthma have an impaired antiviral response, which potentially triggers asthma and worsens COVID-19 progression. Previous studies indicated that interferon production by bronchial epithelial cells and plasmacytoid dendritic cells is deficient in asthmatics.^23^, ^24^, ^25^ It is difficult to estimate how this impaired antiviral immunity influences the course of COVID-19 because severe cases of COVID-19 were reported to be related to overactive immune responses such as cytokine storms.^26^ However, the impaired innate immune response may dampen host protection against SARS-CoV-2 and could result in severe COVID-19. In addition, the possible mechanism of poor prognosis of COVID-19 in asthma is asthma exacerbation caused by SARS-CoV-2. The link between respiratory viral infection and asthma exacerbation is well established.^27^ Rhinovirus is the most commonly associated virus, but other viruses, including coronavirus are also identified, although less frequently.^28^^,^^29^ As with other coronaviruses, SARS-CoV-2 is anticipated to similarly exacerbate asthma, but it is uncertain whether the virus can aggravate asthma itself or bronchoconstriction. The precise pathogenic mechanism of exacerbation due to SARS-CoV-2 has not yet been clearly characterized, but the pneumonic manifestations of COVID-19 may have a more critical influence on patients with chronic airway diseases, such as asthma, because they have less pulmonary reservoirs than normal healthy individuals.

Our study also indicates a higher risk of severe outcomes of COVID-19 in patients with asthma having a recent history of exacerbation of emergency room visits within one year. These data implicate the importance of asthma control in mitigating the risk of severe COVID-19. In the daily practice of patients with asthma, ICS are the main stem of pharmacologic treatment to achieve and maintain asthma control. Corticosteroid use might affect the outcome of COVID-19, but it is not determined whether corticosteroids, either inhaled or systemic, are beneficial or harmful for COVID-19. There is a general concern that corticosteroids may induce suppression of the host immunologic defense response to the virus, which could be related to the severity of COVID-19. However, the use of ICS was associated with lower expression of ACE2 and TMPRSS2, suggesting a beneficial role of ICS in COVID-19.^17^ Furthermore, several reports suggested that ciclesonide may have an inhibitory effect on the replication and activity of SARS-CoV-2 and have potential therapeutic effects on COVID-19.^30^, ^31^, ^32^, ^33^ Although the immunologic effect of asthma medications, including ICS, leukotriene antagonist, and biologics on COVID-19, is not clearly determined, better asthma control using these medications would have more preventive effects on progression to severe COVID-19 and exacerbation of asthma.

Several studies for association between asthma and COVID-19 in Korean population have been reported.^34^, ^35^, ^36^ One study evaluated whether asthma and allergic diseases were associated with an increased likelihood of COVID-19 test positivity and severity. This study revealed non-allergic asthma confers a greater risk of COIVD-19 infection and worse clinical outcomes compared to allergic asthma.^34^ Other studies using Korean national medical claim data of patients with COVID-19 were reported.^35^^,^^36^ Choi et al evaluated the outcomes of COVID-19 including mortality, ICU admission, admission duration, and medical cost after classifying asthma severity using the prescribed asthma medication. After multivariate analyses, they concluded underlying asthma and asthma severity were not independent factors for poor clinical outcomes generally.^35^ Another report studied by Lee et al also reported that asthma patients who had experienced acute exacerbation in the previous year showed higher mortality of COVID-19, but asthma was not a risk factor for poor prognosis of COVID-19.^36^ Unlike these studies, we found the asthma morbidity led to poor prognosis of COVID-19 even after multivariate analysis. Different working definition of asthma and different methods of outcome evaluation of COVID-19 may affect the discrepancy of studies. We included a wider range of subjects with asthma who had a history of asthma symptoms and medication in the last 3 years. A strength of our study differentiated from other studies was the grading system of COVID-19 severity using the demand of oxygen supply and mechanical ventilation. We also performed survival analysis of mortality and subgroup analysis in the elderly to validate our results. Further investigations are needed to elucidate the association between asthma morbidity and COVID-19 severity in larger different populational data.

Our study has several limitations. First, the patients in our study had a confirmed diagnosis for COVID-19, limiting our evaluation for the association between asthma morbidity and susceptibility to COVID-19. Second, we could not evaluate the detailed clinical characteristics, phenotypes, and severity of asthma in the patients with asthma because the claim data did not include symptom control status, laboratory findings, and lung functions. Instead, we assessed the asthma exacerbation history of patients requiring emergency room visits to evaluate the effect of asthma status on the outcome of COVID-19. Asthma phenotypes such as allergic vs. nonallergic phenotype, eosinophilic vs. non-eosinophilic phenotype, and childhood-onset vs. late adult-onset phenotype may affect the outcome of COVID-19. The link between the asthma phenotype and COVID-19 would be an interesting topic for further studies. Third, the detailed clinical features of COVID-19 were not fully evaluated except for their severity in terms of oxygen demand and respiratory support. The features of upper and lower respiratory symptoms, imaging findings of pneumonia, and changes in asthma control status were not assessed in this study using claim data. It is still unclear whether respiratory distress in patients with severe COVID-19 and asthma comorbidity is caused by asthma exacerbation or pneumonia induced by SARS-CoV-2. Studies dealing with changes in asthma due to COVID-19 infection in the future are warranted.

Nevertheless, our study provides evidence of poor prognosis of COVID-19 in patients with asthma and reveals that recent asthma exacerbation could be a risk factor for grave outcomes of COVID-19. In this era of the COVID-19 pandemic, patients with asthma should pay more attention to controlling asthma and adhere to their treatments to avoid poor outcomes. Further studies on the risk of COVID-19, according to asthma phenotypes and detailed characteristics, are warranted.

## Abbreviations

ACE2, angiotensin-converting enzyme 2; CCI, Charlson Comorbidity Index; COVID-19, coronavirus disease; ICS, inhaled corticosteroids; IFN, interferon; LABA, long-acting β2 agonists; NHIS, National Health Insurance Service; SARS-CoV-2, severe acute respiratory syndrome coronavirus 2

## Author contributions

SA and YSC conceived the study. SHK, EJ, SHW, JC, YHK, SA and YSC substantially contributed design of the study, data analysis, and interpretation. SHK, EJ, SA and YSC had full access to all of the data taking responsibility for the accuracy of data analysis and drafted the article. All the authors were involved in the writing and editing of the manuscript and approved the final version to be published.

## Consent for publication

We hereby declare that we all participated in the study and in the development of the manuscript titled “Association of asthma comorbidity with poor prognosis of coronavirus disease 2019”. We have read the final version and give our consent for the article to be published in *the World Allergy Organization Journal*.

## Availability of data and materials

The data supporting the findings of this study are available on request from the corresponding author. The data are not available publicly due to Korean NHIS policy. Approvals from IRB and Korean NHIS are required for data release.

## Ethics approval

The study was approved by the Institutional Review Board of Seoul National University Bundang Hospital (IRB no. X-2004-608-904). The requirement for informed consent was waived under the approval of the IRB.

## Funding

This study was supported by grant no.14-2020-020 from the 10.13039/100016275SNUBH Research Fund.

## Declaration of competing interest

The authors have no conflict of interest to declare.
